# Cooperation Between Aflatoxin-Induced p53 Aberrations and Hepatitis B Virus in Hepatocellular Carcinoma

**DOI:** 10.3390/jox15040096

**Published:** 2025-06-20

**Authors:** Carolina Moreno-León, Francisco Aguayo

**Affiliations:** Laboratorio de Oncovirología, Departamento de Ciencias Biomédicas, Facultad de Medicina, Universidad de Tarapacá, Arica 1000000, Chile; carolinajohanamoreno@gmail.com

**Keywords:** aflatoxin B_1_, p53, TP53 mutation, R249S, hepatocellular carcinoma, HBV, HBx, synergism, carcinogenesis

## Abstract

Hepatocellular carcinoma (HCC) imposes a significant burden on global public health. Exposure to aflatoxins, potent mycotoxins produced by Aspergillus fungi contaminating staple foods, and chronic hepatitis B virus (HBV) infection are major etiological factors, especially where they co-exist. This review examines the critical role of the p53 tumor suppressor pathway as a primary target and convergence point for the carcinogenic actions of aflatoxins and HBV. Aflatoxin B_1_ (AFB_1_), a Group 1 carcinogen, exerts significant genotoxicity, characteristically inducing a specific hotspot mutation (R249S) in the TP53 gene via DNA adduct formation, thereby compromising p53’s critical tumor suppressor functions. This R249S mutation is considered a molecular fingerprint of aflatoxin exposure. Concurrently, the HBV X protein (HBx) functionally inactivates wild-type p53 through direct binding and by promoting its degradation. The synergistic disruption of the p53 pathway, driven by AFB_1_-induced mutation and amplified by HBV-mediated functional inhibition, significantly enhances the risk of HCC development. This review addresses how aflatoxin exposure alters key aspects of p53 and how this damage interacts with HBV-mediated p53 suppression, providing crucial insights into hepatocarcinogenesis. The knowledge synthesized here underscores the importance of mitigating aflatoxin exposure alongside HBV control for effective HCC prevention and treatment strategies.

## 1. The Convergence of HBV, Aflatoxin, and p53 in HCC

Cancer remains a major cause of mortality and reduced life expectancy globally [1]. The development of malignancy is understood as a multistep process involving the acquisition of genetic and epigenetic alterations that drive uncontrolled cell proliferation, evasion of cell death, and eventual metastasis [2,3,4]. Hepatocellular carcinoma (HCC) exemplifies this complexity, ranking as the sixth most common cancer globally and the third leading cause of cancer-related mortality, responsible for hundreds of thousands of deaths annually [5]. In 2022 alone, liver cancer caused approximately 750,000 related deaths worldwide [6]. This highlights HCC as a major global health concern. While HCC etiology is diverse, exposure to dietary aflatoxin B1 (AFB_1_) and chronic infection with the hepatitis B virus (HBV) are recognized as predominant drivers, particularly in specific geographic regions of East Asia and Sub-Saharan Africa, where exposure to both agents is common [7,8,9]. This review will focus on the critical interplay between these two factors, particularly their convergent impact on the p53 tumor suppressor pathway, a key element in hepatocarcinogenesis. HBV infection alone is the most significant risk factor, accounting for about 50% of HCC incidence, while aflatoxin exposure is estimated to contribute to 4.6–28.2% of all global HCC cases [9]. AFB_1_, a potent mycotoxin produced by *Aspergillus* fungi contaminating staple crops like maize and peanuts [10,11,12,13], is classified as a Group 1 human carcinogen by IARC due to its strong association with liver cancer [14]. Chronic HBV infection promotes liver damage through persistent inflammation and viral protein activity, contributing to cirrhosis and creating a pro-carcinogenic environment; HBV can also integrate into the host genome, causing genetic instability and cancer-related gene mutations, further contributing to genetic instability [7,15]. Epidemiological evidence consistently suggests that co-exposure to AFB_1_ and HBV results in a synergistic increase in HCC risk, often exceeding the additive effects of each factor alone [16,17,18]. A key molecular mechanism underlying this synergy involves the convergence of both agents’ detrimental effects on the p53 tumor suppressor pathway. AFB_1_’s significant genotoxicity leads characteristically to a specific mutation in the TP53 gene (R249S) [19,20], while the multifunctional HBV X protein (HBx) directly interacts with and functionally inhibits the wild-type p53 protein [21,22,23,24]. This review focuses specifically on the central role of p53 pathway disruption driven by the interplay between potent aflatoxin-induced mutagenesis and HBV infection in the molecular pathogenesis of HCC.

Extensive epidemiological data from diverse populations provide compelling real-world evidence for the critical role of aflatoxin-induced p53 alterations, particularly in conjunction with HBV infection, in driving HCC [18,25,26,27,28]. The frequency of the specific TP53 R249S mutation in HCC cases exhibits significant geographical variation, strongly correlated with regional levels of AFB_1_ exposure assessed through dietary surveys or biomarker analysis [29,30,31]. This mutation is particularly prevalent in HCC from areas of Sub-Saharan Africa and Southeast Asia, where aflatoxin contamination of staple foods is high, and where HBV infection is often also endemic [19,32,33]. Numerous studies have directly examined the relationship between HBV infection (HBsAg status), quantitative measures of AFB_1_ exposure (e.g., aflatoxin–albumin adducts in serum), and the presence of the R249S mutation. A consistent finding is the significantly increased HCC risk among individuals with evidence of both HBV infection and significant aflatoxin exposure, demonstrating a synergistic effect where the combined risk is substantially greater than the sum of the individual risks [9,25]. Studies measuring AFB_1_ exposure biomarkers have directly linked higher adduct levels to increased HCC risk, particularly among chronic HBV carriers [34]. Furthermore, the R249S mutation itself is frequently detected in individuals with dual exposure [30,32,35]. Its detection in circulating DNA from asymptomatic individuals suggests it can be an early event related to exposure [36]. Research in The Gambia associated aflatoxin exposure markers and the plasma R249S mutation with the risk of liver cirrhosis, a major HCC precursor, noting interactions with HBV status [37]. Moreover, the presence of TP53 mutations, heavily influenced by the R249S prevalence in relevant regions, alongside HBV/AFB_1_ status, independently predicts clinical outcomes such as postoperative tumor recurrence [38]. Conversely, the near absence of the R249S mutation in HCC from populations with low aflatoxin exposure (e.g., Mexico), even if HBV or other risk factors like HCV/alcohol are present [39], strongly reinforces the specific causal link between AFB_1_ exposure and this p53 alteration. These epidemiological findings collectively highlight aflatoxin’s potent role in mutating p53 and establish this event as a key factor mediating the synergy with HBV in hepatocarcinogenesis.

## 2. The p53 Tumor Suppressor Pathway

The p53 protein, encoded by the TP53 gene, functions as a central node in the cellular stress response network, acting as a critical barrier against neoplastic transformation. Often termed the “guardian of the genome,” p53 integrates signals from diverse cellular insults, including DNA damage (such as double-strand breaks, adducts, or stalled replication forks), oncogene activation, hypoxia, ribosomal stress, and nutrient deprivation. Its primary role is to safeguard genomic integrity [40]. Under normal, unstressed conditions, p53 protein levels are kept low through continuous degradation, primarily mediated by the E3 ubiquitin ligase MDM2. Upon stress, p53 is stabilized and activated through post-translational modifications, notably phosphorylation by upstream kinases like ATM and ATR, which sense DNA damage. Activated p53 accumulates in the nucleus, where it functions primarily as a transcription factor, regulating a large network of target genes by activating or repressing them [41]. The cellular outcomes of p53 activation are context-dependent but typically involve inducing cell cycle arrest, senescence, or apoptosis. Cell cycle arrest, frequently mediated by transcriptional upregulation of the CDK inhibitor p21 (CDKN1A), occurs mainly at the G1/S or G2/M transitions, providing a window for DNA repair. If the damage is deemed irreparable, p53 promotes apoptosis by inducing the expression of pro-apoptotic proteins, including Bax, Puma, and Noxa, thereby eliminating potentially dangerous cells. This apoptotic function is extensively studied and crucial for resisting transformation and determining cancer therapy efficacy [42]. p53 also directly influences DNA repair pathways and contributes to maintaining overall genomic stability. Beyond these core functions, p53 is closely integrated with major cellular signaling pathways. It often acts antagonistically to pro-survival and pro-proliferative pathways like the PI3K/Akt/mTOR cascade; for instance, p53 can induce the expression of PTEN, a negative regulator of PI3K/Akt signaling. p53 activity is also modulated by these pathways; Akt can phosphorylate MDM2, enhancing its ability to degrade p53, creating complex feedback loops. Furthermore, p53 signaling crosstalk with pathways regulating inflammation (like NF-κB), angiogenesis (e.g., via thrombospondin-1), and cellular metabolism (regulating glycolysis and oxidative phosphorylation). Therefore, the loss or inactivation of p53 removes not only direct tumor-suppressive functions like apoptosis and cell cycle arrest but also disrupts the balance of these critical signaling networks, significantly contributing to cancer development and progression. Mutations affecting p53’s DNA binding domain are common in cancer, leading to the loss of its tumor-suppressor function and promoting tumorigenesis [43].

## 3. Aflatoxin B_1_-Induced TP53 R249S Mutation: A Molecular Signature in HCC

**Figure 1:**
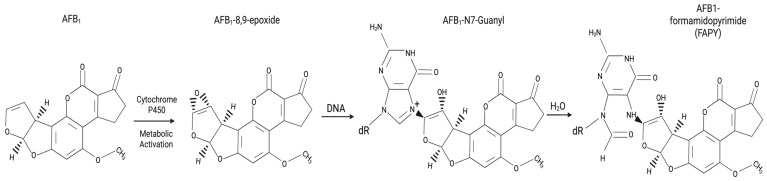
Biotransformation of aflatoxin B1 (AFB_1_) and DNA adduct formation leading to mutagenesis. The metabolic activation of AFB_1_ occurs primarily via cytochrome P450 enzymes (notably CYP1A2 and CYP3A4), producing the highly reactive AFB_1_-8,9-epoxide intermediate. This electrophilic metabolite covalently binds to DNA, preferentially at the N7 position of guanine bases, forming the unstable AFB_1_-N7-Guanine adduct. Subsequent hydrolysis or ring-opening rearrangement of this adduct yields a more persistent and mutagenic lesion, known as the AFB_1_-formamidopyrimidine (FAPY) adduct. These bulky DNA lesions, if not repaired, can induce G→T transversions during replication, notably at codon 249 of the TP53 gene, contributing to the characteristic R249S mutation observed in AFB_1_-related hepatocellular carcinoma.

Aflatoxins are toxic secondary metabolites from Aspergillus species, posing a significant threat through contamination of major food staples worldwide (Figure 1) [44]. Aflatoxin B_1_ (AFB_1_) stands out as the most potent hepatocarcinogen among them [45], causally linked to a substantial proportion of global HCC cases, particularly in high-exposure regions [9,46]. AFB_1_ exerts its significant carcinogenic effect primarily through its genotoxicity following metabolic activation within the liver. Hepatic cytochrome P450 enzymes convert AFB_1_ into the highly reactive AFB_1_-8,9-exo-epoxide [20]. This electrophilic intermediate readily forms covalent adducts with cellular DNA, predominantly at the N7 atom of guanine residues, forming the bulky AFB_1_-N7-Gua lesion [47]. Although cellular DNA repair mechanisms, such as nucleotide excision repair, attempt to remove these adducts, their persistence or inaccurate processing during DNA replication leads to mutations, characteristically G-to-T transversions [48,49,50,51].

A defining feature of AFB_1_-induced mutagenesis in human liver cancer is the high frequency of this specific G>T transversion occurring at the third position of codon 249 (AGG → AGT) within the TP53 gene [19]. This mutation, accounting for up to 90% of TP53 mutations in AFB_1_-related HCC, encodes an arginine residue essential for p53’s DNA binding ability [52]. The resulting R249S amino acid substitution is widely recognized as a molecular signature or fingerprint of significant AFB_1_ exposure and is frequently detected (sometimes in >50% of tumors) in HCC cases originating from regions with high levels of dietary aflatoxin contamination [30]. This mutation accounts for up to 90% of TP53 mutations in AFB_1_-related HCC [53]. The R249S mutation represents a critical loss-of-function event, as the altered protein fails to effectively bind to p53 DNA response elements and activate downstream target genes necessary for cell cycle arrest and apoptosis [54]. Notably, evidence indicates that the R249S mutant is not merely inactive but acquires distinct oncogenic gain-of-function properties. These pro-tumorigenic activities may include enhancing the transcription of growth-promoting factors like IGF-II [55], altering interactions with crucial transcription factors such as Sp1 [55], and providing resistance to apoptotic signals, including those triggered by TNF-alpha or the HBV HBx protein [56,57]. Emerging research suggests complex mechanisms for this gain of function, potentially involving interactions with other proteins like CDK4/cyclin D1 and PIN1 or forming complexes with TANK-binding protein kinase 1 to evade immune surveillance [58]. These acquired functions likely confer a selective growth and survival advantage upon hepatocytes harboring the mutation, particularly in the context of chronic liver inflammation typical of high-risk settings [59]. The specific targeting of TP53 codon 249 by AFB_1_ provides a direct mechanistic link between this significant environmental carcinogen and the disabling of a key tumor suppressor pathway in the liver [60].

## 4. Hepatitis B Virus and HBx-Mediated Disruption of p53 Function

Hepatitis B virus (HBV) is an enveloped DNA virus, characterized by a small genome of approximately 3.2 kb. While its structure is defined by these features, its replication occurs in the cytoplasm through the reverse transcription of an encapsidated pre-genomic RNA (pgRNA) into a partially double-stranded relaxed circular DNA (RC-DNA) [61]. Chronic HBV infection is a major driver of HCC, involving multiple processes to promote liver cell transformation [62,63]. Besides inducing chronic inflammation and integrating its DNA into the host genome [64], HBV actively interferes with cellular tumor suppressor mechanisms, including the p53 pathway. This interference is largely mediated by the HBV X protein (HBx), a small (154-amino acid) regulatory protein with pleiotropic functions critical for the viral life cycle and implicated in hepatocarcinogenesis [65]. HBx is produced in about 70% of HBV-associated HCC [66]. HBx contributes to the inhibition of wild-type p53 through several distinct mechanisms. Firstly, HBx can physically interact with the p53 protein. This binding, often involving the C-terminal domain of p53, hinders p53’s ability to bind its target DNA sequences, thereby blocking its function as a transcriptional activator of tumor suppressor genes [21]. This direct interaction inhibits p53’s response element-directed transactivation [67]. Secondly, HBx promotes the degradation of the p53 protein. It facilitates this by upregulating or enhancing the activity of MDM2, the primary E3 ubiquitin ligase that targets p53 for destruction via the proteasome [68]. Another proposed mechanism involves HBx sequestering p53 in the cytoplasm, reducing the nuclear concentration available to trigger apoptosis [69]. This effect might be linked to HBx’s ability to activate signaling pathways like NF-κB [70,71]. By reducing the stability and thus the intracellular levels of functional p53, HBx diminishes the cell’s capacity to respond appropriately to stress signals. Thirdly, HBx can suppress p53-mediated apoptosis [21], allowing infected cells potentially harboring DNA damage or viral integration events to survive and proliferate. While HBV integration events can cause genomic instability [62,72], the concurrent functional inactivation of p53 by HBx is likely crucial for preventing the elimination of these genetically compromised cells. Additionally, HBx contributes to a pro-oncogenic cellular environment through the induction of oxidative stress and the activation of various pro-survival signaling pathways (e.g., PI3K/Akt/mTOR, Wnt, and Notch) [64,73,74,75], which can further counteract or bypass p53-dependent tumor suppression. Interestingly, HBx has also been shown to interact with and inactivate TAp63, a p53 homolog that can suppress HCC growth in cells with defective p53 [76].

## 5. Synergistic Disruption of p53 by Aflatoxin-Induced Mutation and HBV Activity

The significantly increased risk of HCC observed in individuals co-exposed to chronic HBV infection and dietary aflatoxins points strongly toward a synergistic interaction between these two carcinogens [16,37]. This synergy manifests prominently through molecular interactions involving the p53 pathway. The HBV–aflatoxin interaction likely results from the combined and complementary attacks on the p53 function. AFB_1_ delivers a direct mutagenic hit, frequently causing the function-altering R249S mutation [19], while HBV/HBx works concurrently to inhibit the function and promote the degradation of any remaining wild-type p53 protein [77]. This dual mechanism results in a more significant and sustained loss of p53-mediated tumor suppression than would likely occur with exposure to either agent alone.

Furthermore, HBx might directly enhance the mutagenic impact of AFB_1_. Experimental studies suggest that HBx expression increases the frequency of the specific AFB1-induced R249S mutation in vitro [78]. This potentiation could arise from HBx impairing the cellular DNA repair pathways responsible for removing the AFB_1_-DNA adducts before they cause mutations, or by inhibiting the apoptotic elimination of cells that have successfully acquired the TP53 mutation, thereby promoting their survival and potential for clonal expansion [66,79,80]. Additionally, functional cooperation between the R249S mutant p53 protein itself and HBx might contribute to the synergy. Studies suggest that p53-R249S and HBx can form a complex, potentially conferring a growth advantage, particularly in early hepatocarcinogenesis [54]. Both possess anti-apoptotic capabilities and their co-expression could confer a significant survival and proliferative advantage, particularly during the crucial early stages of hepatocarcinogenesis [54,81].

**Figure 2:**
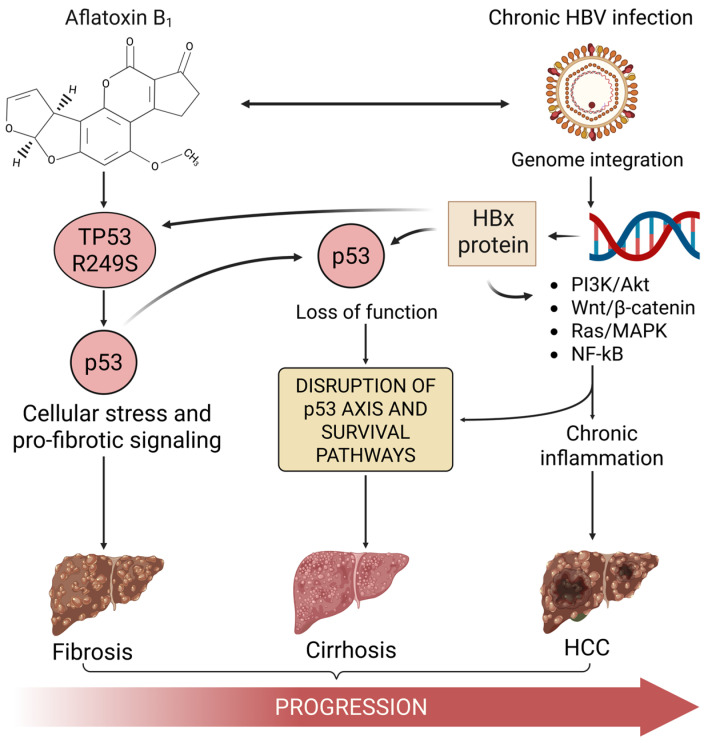
Cooperative mechanisms by which aflatoxin B_1_ (AFB_1_) and chronic hepatitis B virus (HBV) infection promote hepatocellular carcinoma (HCC) through p53 pathway disruption. AFB_1_ is metabolically activated in the liver and induces the hotspot TP53 R249S mutation, resulting in p53 downregulation. This loss of p53 function contributes to cellular stress and pro-fibrotic signaling, leading to fibrosis. Independently, chronic HBV infection integrates into the host genome and expresses the HBx protein, which functionally suppresses p53 and activates oncogenic signaling pathways including PI3K/Akt, Wnt/β-catenin, Ras/MAPK, and NF-κB. These signals promote chronic inflammation and further impair apoptosis and cell cycle regulation. The combined effects converge on the disruption of the p53 axis and cellular survival pathways, facilitating the pathological progression from fibrosis to cirrhosis and ultimately HCC.

The consequence of this significant p53 pathway disruption, driven by both aflatoxin-induced mutation and HBV/HBx activity, is a compromised cellular response to DNA damage. This failure allows the genomic instability, fueled by both the continuous formation of AFB_1_ adducts and HBV-related factors (integration, chronic inflammation, and oxidative stress), to accelerate the accumulation of additional genetic and chromosomal alterations [30]. This heightened genomic instability, occurring in cells that have lost their p53 safeguard, contributes to the multistep progression toward malignant HCC [82]. Figure 2 addresses the role of both AFB_1_ and HBV in promoting p53 downregulation, fibrosis, cirrhosis, and HCC. In addition, it includes the major signaling pathways involved in liver cancer pathogenesis, including Wnt, PI3K-AKT-STAT3, Ras-MAPK1, and NF-κB. These are commonly activated during liver tumor development and are frequently modulated by HBV infection and aflatoxin exposure [14,83].

## 6. Conclusions: Aflatoxin-Induced p53 Mutation as a Key Driver in HBV-Associated HCC

In conclusion, the p53 tumor suppressor pathway is a central molecular target significantly compromised by both dietary aflatoxin exposure and chronic HBV infection, explaining their strong synergistic interaction in driving hepatocellular carcinoma. Aflatoxin B_1_, a widespread environmental carcinogen, directly damages DNA and induces a characteristic R249S mutation in the TP53 gene, a key molecular event that impairs p53’s protective functions and may confer oncogenic properties. HBV infection, particularly through the actions of the HBx protein, further undermines the p53 pathway by inhibiting wild-type p53 activity and promoting its degradation. This combined attack—specific mutation by AFB_1_ coupled with functional inactivation by HBV, leads to a significant disruption of cellular anti-oncogenic mechanisms.

The resulting synergistic loss of p53 function provides a strong molecular explanation for the significantly elevated HCC risk observed in co-exposed populations globally. Understanding this interplay, particularly the important role of aflatoxin in inducing the specific p53 mutation that is then amplified in the context of HBV infection, is crucial for public health. It underscores the critical need for comprehensive prevention strategies targeting both a reduction in aflatoxin exposure in food supplies and widespread HBV vaccination and treatment. The R249S mutation stands as a valuable biomarker of aflatoxin-related genetic damage and HCC risk. While HCC pathogenesis is multifaceted, the convergent disruption of the p53 pathway, initiated significantly by aflatoxin mutagenesis, represents a key mechanism underlying the significant carcinogenicity associated with combined HBV and aflatoxin exposure, demanding continued research and targeted interventions.

Future research should focus on understanding how aflatoxin-induced p53 mutations and HBV infection work together to drive liver cancer. Indeed, more studies are needed to clarify how the HBx protein may increase the mutagenic impact of AFB1, possibly by interfering with DNA repair or by protecting mutated cells from being eliminated. Exploring the combined effects of the TP53 R249S mutation and HBx could also help uncover new mechanisms that give cancer cells a survival advantage and identify potential targets for therapy. From a public health standpoint, efforts should be directed toward using the R249S mutation and HBx expression as early biomarkers for liver cancer risk, especially in regions with high aflatoxin exposure and HBV prevalence. At the same time, reinforcing preventive strategies such as HBV vaccination, antiviral treatment, and strict control of aflatoxin contamination in food can significantly reduce HCC incidence. These combined approaches should be evaluated in real-world settings to guide effective cancer prevention programs.

## Data Availability

No new data were created or analyzed in this study.
